# Monitoring of Antipsychotics in CAMHS Intellectual Disability Service in Lancashire and South Cumbria NHS Foundation Trust

**DOI:** 10.1192/bjo.2024.542

**Published:** 2024-08-01

**Authors:** Sadia Batool, Wala Kamal Abbas, Abimbola Oyedokun, Mischa Oyed

**Affiliations:** 1 LSCFT, Preston, United Kingdom; 2 CWP, Cheshire, United Kingdom

## Abstract

**Aims:**

To ascertain the service performance against the standards set by National Institute for Clinical Excellence (NICE) guidelines on physical health monitoring of children and adolescents prescribed antipsychotics.

**Methods:**

Initial audit: April–June 2021.

Re-audit: January–February 2024.

Registered with the Lancashire and South Cumbria NHS Foundation Trust audit department. An audit tool was developed by the investigators. The investigators carried out a review of patient electronic records and clinical letters to gather information pertaining to initiation of antipsychotics and physical health monitoring.

**Results:**

Amongst other variables in this trust-wide audit, we considered age, ethnic background, reason of initiation of anti-psychotic, comorbid conditions among which most common is epilepsy, dose of antipsychotic used and distributions across various CCGs. Were they regularly reviewed by medic reviews and side effects monitored? We also looked at choice of antipsychotic used, which was largely aripiprazole and risperidone. Were antipsychotic bloods done or not and were we able to complete children's height and weight measurements whilst they were on antipsychotics? It was important that these are documented as being considered or ‘offered' even if could not be successfully completed due to e.g. challenging behaviour from the child. Detailed and comparative results can be shown in final submission along with charts.

**Conclusion:**

The recommendations from initial audit were compared with the second audit, and whilst some of them were completed such as incorporating growth chart in the electronic records system, some ongoing challenges were identified. Positive and negative findings were both noted although the final conclusions lies in favour of good changes been made to service including the caseload becoming more ID specific in this age group.

